# Ammonia-Responsive Gelatin/Co–MOF Composite Films Based on Gallic Acid-Derived Metal–Organic Frameworks for Intelligent Food Packaging

**DOI:** 10.3390/polym18161938

**Published:** 2026-08-07

**Authors:** Mahmut Ekrem Parlak, Burcu Demirtaş, Ayse Neslihan Dundar, Oya Irmak Sahin, Adnan Fatih Dagdelen, Furkan Turker Saricaoglu, Luca Rastrelli, Maria D’Elia, Sadettin Turhan

**Affiliations:** 1 Department of Food Engineering, Faculty of Engineering and Natural Science, Bursa Technical University, 16310 Bursa, Türkiye; ekrem.parlak@btu.edu.tr (M.E.P.); burcudemirtas.bd50@gmail.com (B.D.); ayse.dundar@btu.edu.tr (A.N.D.); adnan.dagdelen@btu.edu.tr (A.F.D.); 2 Department of Chemical Engineering, Faculty of Engineering, Yalova University, 77200 Yalova, Türkiye; oyairmak.sahin@yalova.edu.tr; 3 Furya Nanofiber Food R&D Engineering Project Consultancy Industrial Trade Company, 16310 Bursa, Türkiye; 4 Department of Pharmacy, University of Salerno, 84084 Salerno, Italy; mdelia@unisa.it; 5 National Biodiversity Future Center (NBFC), 90133 Palermo, Italy; 6 Dipartimento di Scienze della Terra e del Mare, University of Palermo, 90133 Palermo, Italy; 7 Department of Food Engineering, Faculty of Engineering, Ondokuz Mayis University, 55200 Samsun, Türkiye; sturhan@omu.edu.tr

**Keywords:** gelatin film, cobalt metal–organic framework, intelligent packaging, ammonia sensing, food freshness monitoring

## Abstract

Ammonia-responsive gelatin-based composite films containing cobalt metal–organic frameworks (Co–MOFs) synthesized using gallic acid as an organic ligand were developed and evaluated as intelligent packaging materials. Co–MOFs were incorporated into gelatin films at concentrations of 2.5, 5.0, 7.5, and 10.0% (*w*/*w*, based on gelatin). The effects of Co–MOF incorporation on the physicochemical, structural, thermal, mechanical, and sensing properties of gelatin films were systematically investigated. Increasing Co–MOF content reduced film moisture content (from 14.47 to 13.25–13.58%) and swelling capacity (from 599.37 to 484.88–547.30%), while increasing solubility (from 39.09 to 48.88%), water vapor permeability (WVP; from 1.652 to 2.054 g·mm/m^2^·h·kPa), and moisture sorption behavior. Sorption isotherm analyses based on the Guggenheim–Anderson–de Boer (GAB) and Brunauer–Emmett–Teller (BET) models confirmed enhanced water adsorption capacity and increased specific surface area in the films (from 356.13 to 455.74 m^2^/g). Scanning electron microscopy (SEM), Fourier transform infrared spectroscopy (FTIR), X-ray diffraction (XRD), and differential scanning calorimetry (DSC) analyses demonstrated successful incorporation of Co–MOFs into the gelatin matrix, revealing good dispersion at low and moderate concentrations and partial aggregation at higher loadings. The incorporation of Co–MOFs improved the thermal stability of the films, while only a moderate reduction in mechanical strength was observed with increasing filler content. The composite films exhibited rapid and concentration-dependent colorimetric responses toward ammonia vapor. After 120 min of exposure, the color difference (ΔE) increased from less than 1 in the control film to approximately 12, 15, 24, and 27 for G/Co–MOF2.5, G/Co–MOF5, G/Co–MOF7.5, and G/Co–MOF10 films, respectively. Films containing higher amounts of Co–MOF showed faster response kinetics and greater color differences, enabling clear visual detection of ammonia. These findings demonstrate that gelatin/Co–MOF composite films based on gallic acid-derived metal–organic frameworks are promising intelligent packaging materials for monitoring food freshness and spoilage through ammonia detection.

## 1. Introduction

In recent years, pH/ammonia-responsive intelligent packaging has emerged as a rapidly growing research area, as it can promptly reflect the freshness or spoilage status of foods such as eggs, dairy products, fish, and meat during storage through visible color changes [1,2,3]. These packaging systems are typically developed by incorporating pH- or ammonia-sensitive compounds into biodegradable biopolymer matrices, including proteins, polysaccharides, or their blends [1,3]. Natural pigments, particularly anthocyanins, have been widely used as colorimetric indicators for smart food packaging. However, their application may be limited by complex extraction procedures, relatively high cost, poor stability under light and heat, and insufficiently distinct color changes in the pH range commonly associated with food spoilage [1,2,3]. Therefore, the development of alternative ammonia-responsive materials with improved stability, easy preparation, and clear visual response remains an important objective in intelligent food packaging.

Metal–organic framework (MOF) nanoparticles have recently attracted increasing attention as colorimetric and functional components for monitoring food freshness and spoilage [4]. MOFs offer several advantageous features, including controllable pore size, high surface area, structural tunability, functional flexibility, and good compatibility with polymer matrices [5]. Their inorganic–organic hybrid structure makes them suitable as nanofillers for the development of polymer-based composite films with improved functional properties [2,6]. Among different MOFs, cobalt-based MOFs (Co–MOFs) are particularly interesting for ammonia sensing because of their structural rigidity, visible coloration, chemical stability, and gas-responsive behavior [4,7,8]. For example, Feng et al. [3] developed Co–MOF-loaded sodium alginate films with ammonia-sensitive discoloration and shrimp freshness monitoring ability. Noori et al. [6] showed that Co–MOF nanoparticles incorporated into polyvinyl alcohol/chitosan nanofiber films enhanced volatile amine detection. Tao et al. [4] also reported cellulose acetate films containing Co–MOF with visible responses toward ethanol, NH_3_, and NH_3_-H_2_S.

The properties of MOFs are strongly influenced not only by the metal ions but also by the organic ligands used during synthesis. Conventional MOF ligands often include aromatic, imidazole-based, amine-containing, or cyanide-containing structures, such as 2-amino-1,4-benzene sulfonic acid, lauric acid, imidazole, and cyanide derivatives [1,3,4,9]. However, the suitability of some ligands for food-contact applications may be limited. For this reason, the use of naturally occurring and food-derived ligands is of particular interest for developing MOF-based materials intended for intelligent food packaging. Gallic acid is a natural polyphenol containing one carboxylic group and three phenolic hydroxyl groups, which can act as coordination sites for metal ions and support the formation of metal–organic networks [10]. Therefore, gallic acid represents a promising ligand for obtaining Co–MOFs with improved suitability for food-related applications.

Biopolymer-based films used in intelligent packaging are commonly prepared from natural polymers such as carboxymethyl cellulose [1], chitosan [2], sodium alginate [3], cellulose acetate [4], agar [8], pectin [11], gelatin and [12] carrageenan [12]. Among these, gelatin is a protein-based hydrocolloid derived from collagen and is widely used in film-forming systems because of its transparency, flexibility, film-forming ability, and gas barrier properties [13,14]. However, gelatin films often show limitations related to moisture sensitivity and mechanical performance. The incorporation of functional nanofillers, including MOFs, may improve or modulate these properties while also providing additional sensing functionality. In our previous study, Co–MOF-containing thermoplastic starch films were developed for freshness monitoring of shrimp [15], suggesting the potential of Co–MOF systems as ammonia-responsive indicators in food packaging.

Although Co–MOF-based films have been investigated for intelligent packaging applications, the use of gallic acid-derived Co–MOFs in gelatin-based films remains insufficiently explored. In particular, limited information is available on how these MOFs affect the physicochemical, structural, thermal, mechanical, water sorption, and ammonia-responsive properties of gelatin films. Moreover, the combined effects of gallic acid-derived Co–MOFs on the moisture sorption behavior, structural characteristics, thermal stability, mechanical performance, and ammonia-sensing properties of gelatin films have not been comprehensively evaluated. The novelty of this study lies in the development of ammonia-responsive gelatin films incorporating Co–MOFs synthesized using gallic acid, a naturally occurring polyphenolic ligand with potential suitability for food-related applications. In addition, this work establishes relationships between Co–MOF loading, moisture sorption behavior, and ammonia responsiveness. Accordingly, this study aimed to develop gelatin-based composite films containing different concentrations of gallic acid-derived Co–MOFs and to evaluate their potential as ammonia-responsive intelligent packaging materials. The films were characterized in terms of physicochemical properties, water vapor permeability, sorption behavior, morphology, optical properties, thermal stability, crystallographic structure, molecular interactions, mechanical performance, and ammonia sensitivity.

## 2. Materials and Methods

### 2.1. Materials

Gelatin from bovine hide (Bloom strength: approximately 225 g) was kindly donated by Bursa Jelatin Inc. (Bursa, Türkiye).

Glycerol (99.5% purity and 1.28 g/mL density) and cobalt nitrate hexahydrate [Co(NO_3_)_2_·6H_2_O], dimethyl formamide (DMF), and gallic acid used for MOFs were also sourced from Sigma-Aldrich (Darmstadt, Germany).

### 2.2. Synthesis of Co–MOFs

The Co–MOFs were synthesized according to the procedure outlined by Feng et al. (2023) [3], with slight modifications. To achieve this, 0.002 moles of Co(NO_3_)_2·_6H_2_O were dissolved in a 1:1 (*v*/*v*) mixture of DMF and water, yielding a total volume of 20 mL. Subsequently, 0.004 moles of gallic acid were introduced into the solution as an organic ligand, followed by stirring for 1 h. The mixture was transferred into a Teflon-lined stainless steel autoclave and heated at 120 °C for 12 h, after which the precipitates were isolated, washed three times with absolute ethanol, and ultimately vacuum-dried at 50 °C. The synthesized Co–MOFs were stored in amber bottles at 4 °C until utilization.

### 2.3. Production of G/Co–MOF Composite Films

Gelatin films were produced using the pouring method, as described in our previous study [16]. In this respect, 5 g of gelatin was added to 100 mL of distilled water and stirred on a magnetic stirrer at 80 °C for 2 h. Then, the mixture was stirred on a magnetic stirrer until it reached room temperature. Co–MOF was then added at concentrations of 2.5% (G/Co–MOF2.5), 5% (G/Co–MOF5), 7.5% (G/Co–MOF7.5), and 10% (G/Co–MOF10) relative to the polymer concentration (0.2, 0.4, 0.6, and 0.8 g) and stirred for an additional 2 h. In this study, the gelatin film sample without Co–MOF was the control sample (G/Co–MOF0). Subsequently, 65 mL of the solution was spread onto 40 cm × 40 cm glass plates covered with mylar film using a drum bar, resulting in a wet thickness of 0.889 mm. After drying overnight at room temperature, the dried film solutions were carefully removed from the surface. The resulting films were subsequently placed in a desiccator containing a saturated Mg(NO_3_)_2_ solution to maintain a humidity of 54% and stored for 3 days.

### 2.4. Physicochemical and Water Vapor Barrier Properties

Film thickness was determined by averaging measurements taken from 20 random locations on each film using a digital micrometer (Mitutoyo, 293-IP-54, Kawasaki, Japan). The film’s moisture content was determined by subjecting film specimens (2 cm × 2 cm) to an oven at 105 °C for 24 h, followed by calculation of the weight loss. The swelling index was determined by immersing dried film specimens in distilled water at 25 °C for 10 s, removing excess water from the film surface with a paper towel, and subsequently calculating the difference between the dry and wet weights [17]. Film solubility was evaluated by immersing the dried films in 30 mL of distilled water at 25 °C for 24 h under magnetic stirring. The undissolved film parts were removed and dried overnight at 105 °C. The weight differences between the initial and final dry weights were then calculated.

Water vapor permeability (WVP) was determined using the modified gravimetric cup method described by McHugh et al. [18]. The circular films, with a diameter of 6 cm, were fixed onto 5 cm diameter poly(methyl methacrylate) cups filled with 6 mL of distilled water. The cups were placed inside a cabinet containing anhydrous silica (0% relative humidity), with air circulation facilitated by a fan. The measurements were performed at 25 ± 1 °C under a relative humidity gradient of 100–0% RH. Water vapor permeating through the film led to water loss from the cups, which was quantified using an analytical balance at 2 h intervals. A total of 8 measurements were taken over 2 days. WVP measurements were conducted in triplicate for each film, and they were calculated as follows:

(1) $$WVP=\frac{dw}{dt}\times \frac{1}{A}\times \frac{L}{∆P\times ∆RH}$$

where WVP is water vapor permeability, w/t is the weight loss rate (g/h), L is film thickness (mm), A is exposed film area (m^2^), and ΔP is the partial water vapor pressure difference across the film.

### 2.5. Sorption Isotherms

A water activity device (Novasina AG, LabMaster, Lachen, Switzerland) was used to determine the moisture sorption isotherms of gelatin-based composite films incorporating Co–MOF. To achieve this, a range of saturated salt solutions with water activity (a_w_) values of 0.047, 0.111, 0.327, 0.531, 0.736, 0.830, and 0.881 were employed. Film specimens (3 cm × 3 cm) were dried in an air-circulated oven at 40 °C overnight. Saturated salt solutions were then transferred to the reference holder, while the film samples were placed in the sample holder cell. Measurements were conducted until the equilibrium water activity was achieved at 25 °C. The equilibrium moisture content (EMC) was calculated as the weight of water per g of dry film by subtracting the weight after equilibrium from the initial dry weight. The EMC–water activity curves were used to analyze sorption isotherms. The moisture sorption data were fitted using two widely adopted models, GAB (Guggenheim–Andersen–de Boer) and BET (Brunauer–Emmett–Teller), as follows:

(2) $$M_{w}=\frac{C\times k\times M_{0}\times a_{w}}{\left[\left(1-k\times a_{w}\right)\times \left(1-k\times a_{w}+c\times k\times a_{w}\right)\right]}$$

(3) $$M_{w}=\frac{C\times M_{0}\times a_{w}}{\left[\left(1-a_{w}\right)\times \left(1+\left(c-1\right)\times a_{w}\right)\right]}$$

where $M_{w}$, $M_{0}$, and $a_{w}$ in these equations represent EMC (g water/g dry film), monolayer moisture content (g water/100 g dry film), and water activity (RH/100), respectively, while C, and k are the model constants. $M_{0}$ values obtained from the GAB model were then used to the calculate specific surface area $\left(S_{0}\right)$ of the films. This value can be defined as the surface area of particles per unit mass of films or per unit volume of particles, including all accessible inner surfaces, particularly pore wall surfaces [19]:

(4) $$S_{0}=M_{0}\times \frac{N_{0}\times A_{H_{2}O}}{M_{H_{2}O}}=M_{0}\times 3.5\times 10^{3}$$

where $N_{0}$ represents the Avogadro’s number, $A_{H_{2}O}$ refers to the molecular area of water (m^2^/mole) and $M_{H_{2}O}$ indicates the molar mass of water (g/mol). Nonlinear regression analysis for fitting the GAB and BET sorption models was performed using OriginPro 2021 software (OriginLab Corporation, Northampton, MA, USA).

The BET and GAB models were selected because they provide complementary information regarding moisture sorption mechanisms. The BET model was applied to estimate monolayer moisture content and evaluate the primary adsorption sites available within the film matrix. The GAB model, which extends the BET theory by incorporating multilayer adsorption effects and water molecule interactions, was used to describe the overall moisture sorption behavior over the entire experimental water activity range. Thus, the combined application of both models enabled a more comprehensive evaluation of water–film interactions and the effect of Co–MOF incorporation on moisture sorption characteristics.

### 2.6. Microstructural Properties

The morphology of G/Co–MOF composite films was evaluated using scanning electron microscopy (SEM, Carl Zeiss, Gemini 300, Oberkochen, Germany). Film cross-sections were obtained by fracturing samples in liquid nitrogen prior to analysis. Images were captured at an accelerating voltage of 5 kV, and magnification was 5000× for Co–MOFs and 1000× for G/Co–MOF films. All samples were coated with gold–palladium at 25 mA using a sputter coater (Leica, EM ACE600, Teaneck, NJ, USA). The elemental distribution at the film surface was analyzed using a SEM equipped with energy-dispersive X-ray spectroscopy (SEM/EDX).

### 2.7. Color, Opacity, and Visual Appearance

A colorimeter (HunterLab, UltraScan VIS, VIR, Reston, VA, USA) was used to directly measure L*, a*, and b* values at five randomly selected surface points on the films. For device calibration, a white standard plate (L* = 96.11, a* = −0.25, b* = 1.06) was used. For the opacity calculation, the absorbance at 600 nm was recorded, following the method outlined by Kurt et al. [20]. The visual appearance of G/Co–MOF films was captured using a camera.

### 2.8. Thermal Properties

Differential scanning calorimetry (TA Instruments, Discovery 251, New Castle, DE, USA) was used to characterize the thermal properties of Co–MOF and G/Co–MOF composite films. To conduct the analysis, dried film samples weighing 4–5 mg were placed on an aluminum pan and examined over a temperature range of −25 to 250 °C. An empty aluminum pan was used as a reference. The Co–MOF was analyzed over a broader temperature range of −25 to 500 °C. The nitrogen gas flow and heating rates were set at 20 mL/min and 5 °C/min, respectively.

### 2.9. Crystallographic Properties

The crystal structure of Co–MOF and G/Co–MOF composite films was determined using a wide-angle X-ray diffraction (XRD) instrument (Bruker, AXZ Discovery D8, Billerica, MA, USA) equipped with a Cu-Kα radiation source (λ = 1.546 Å). The X-ray source was operated at 40 kV and 40 mA. The incidence angle 2θ ranged from 4 to 40° at a scanning rate of 2° min^−1^. All samples were stored in a desiccator containing anhydrous silica for 3 weeks to eliminate moisture.

### 2.10. Molecular Characterization by FTIR

FTIR (Fourier transform infrared spectroscopy) spectra of Co–MOF and G/Co–MOF composite films were obtained using an infrared spectrometer (Bruker, Alpha II, Fällanden, Switzerland) equipped with an attenuated total reflectance (ATR) accessory over 400–4000 cm^−1^, with a spectral resolution of 4 cm^−1^. Each spectrum was collected from 32 scans. Before the measurements, all samples were dried in a desiccator containing anhydrous silica for 3 weeks.

### 2.11. Mechanical Properties

Tensile strength (TS) and elongation at break (EAB) were determined according to ASTM D882-26, Standard Test Method for Tensile Properties of Thin Plastic Sheeting [21]. Film specimens (8 cm length × 1 cm width) were cut with a razor blade, and the tensile grips of the device (TA-HD Plus, Stable Micro Systems, Godalming, UK) were fixed. The distance between the tensile grips was maintained at 6 cm while the testing speed was fixed at 2 mm/s. Prior to testing, films were conditioned at 25 °C and 54% RH for at least 48 h. During these tests, the stress–strain curves obtained were subsequently used to determine the TS and EAB for each film sample. Each film was evaluated at least 8 times. Additionally, the puncture force (PF) and puncture deformation (PD) of the film samples were also investigated. For this, the films were cut into 2 cm by 2 cm rectangular strips and placed into a porous support ring with an internal diameter of 10 mm. A centrally located 6.25 mm ball probe was pushed through the ring at a fixture speed of 0.2 mm/s. The PF and PD values were recorded and presented in the experimental results, generated using the testing device’s software-based strain/stress curves. All samples underwent testing at least three times to ensure accuracy and reliability.

### 2.12. Ammonia Sensitivity of Films

The color change (Δ*E*) on G/Co–MOF films upon exposure to ammonia vapor was measured using a colorimeter (HunterLab, UltraScan VIS, VIR, USA). To conduct the analysis, the films were cut into 4 cm × 3.5 cm and fixed to the edges of 25 mL color measurement containers. Subsequently, 100 ppm of a 25% ammonia solution was added and positioned 3 cm below the films. The lid of the container was tightly sealed with Parafilm, and the ΔE of the films was monitored at 2 min intervals over 120 min [3]. ΔE of the films was calculated as follows:

(5) $$∆E=\sqrt{{\left(L_{0}-L^{*}\right)}^{2}+{\left(a_{0}-a^{*}\right)}^{2}+{\left(b_{0}-b^{*}\right)}^{2}}$$

where the subscript “o” designates the color measurement of a control sample utilized as the reference point. A higher ∆*E* value signifies a more pronounced color variation from the reference sample.

### 2.13. Statistical Analysis

The collected data from the physicochemical, elemental, color/optical, and mechanical analyses of the G/Co–MOF composite films were subjected to one-way analysis of variance (ANOVA) using the SPSS software (IBM SPSS Statistics, v22, IBM Inc., Armonk, NY, USA). Significant differences between means were evaluated using Duncan’s multiple-range test at the 0.05 significance level. All the data are presented as the mean ± SD. All experiments were conducted at least in triplicate unless otherwise stated.

## 3. Results and Discussion

### 3.1. Physicochemical and Water Vapor Barrier Properties

Physicochemical properties, such as thickness, swelling index, and solubility, as well as barrier performance, are essential for the development of smart packaging materials. The physicochemical and water vapor barrier properties of G/Co–MOF composite films containing Co–MOF at different concentrations are shown in Table 1. The thickness of gelatin film (G/Co–MOF0), which was 0.057 mm, decreased to 0.030–0.045 mm when Co–MOF was added (*p* < 0.05). Since all films were prepared using the same volume of film-forming solution and dried under identical conditions, the observed reduction in thickness cannot be attributed to differences in casting volume or drying conditions. This decrease in thickness may be associated with interactions between Co–MOF particles and the gelatin matrix, which contributed to a more compact film structure. Hydrogen bonding and other intermolecular interactions between functional groups of gelatin chains and the surface of gallic acid-derived Co–MOF structures may facilitate closer polymer chain arrangement and decrease the free volume within the matrix, resulting in thinner films. The moisture content of the gelatin and G/Co–MOF composite films also supports these results. As shown, all G/Co–MOF composite films containing varying concentrations of Co–MOF exhibited lower moisture content than the gelatin film (*p* < 0.05). This indicates that gallic acid, used as an organic ligand, may introduce hydrophobic groups via covalent cross-linking, thereby reducing moisture content. Similar observations were also reported in the incorporation of L-malic acid/Co–MOF in pectin–gelatin matrices to develop versatile food preservation composite films [22]. The swelling index of the films ranged from 484.88 to 599.37%. G/Co–MOF composite films, especially those containing high concentrations of Co–MOF, exhibited a lower swelling index than the gelatin control film, suggesting that the incorporation of Co–MOF can influence the films’ water-absorption properties. However, the swelling index did not show a continuous decrease with increasing Co–MOF concentration, indicating that the swelling behavior was affected by multiple factors. This variation may be attributed to the balance between the water affinity of Co–MOF structures and the interactions between Co–MOF particles and the gelatin matrix. The incorporation of Co–MOF may enhance polymer–filler interactions and promote a more compact matrix structure, thereby limiting water penetration; however, changes in filler distribution at different concentrations may contribute to the observed fluctuations in swelling behavior.

While the control film without Co–MOF demonstrated a solubility of 39.09%, the solubility of composite films notably increased (*p* < 0.05) in a concentration-dependent manner with the addition of Co–MOF and reached the highest value of 48.88% in the G/Co–MOF10 film.

Since MOFs are characterized by high surface area and porous structures [23,24], their incorporation may increase the interaction between water molecules and the composite matrix, thereby contributing to the observed increase in film solubility.

These findings are also consistent with those of Weiss et al. [25] and Yilmaz et al. [15], who reported that smaller particle sizes correspond to larger surface areas and, consequently, to increased solubility.

The water vapor barrier properties of composite films are known to be influenced by the type and concentration of nanofillers and the polymer matrix [11,26]. As with solubility, the lowest WVP was 1.652 g·mm/m^2^·h·kPa in the control film without Co–MOF, and the addition of Co–MOF gradually increased the WVP of the composite films in a concentration-dependent manner (*p* < 0.05). This increase may be attributed to the porous nature of Co–MOF particles, which can facilitate the transfer of water molecules through the composite film structure [23]. Similarly, Yilmaz et al. [15] and Ingole et al. [27] and reported that when MOF nanoparticles were incorporated into a polymer matrix, the surface area of thin-film nanocomposite membranes increased, leading to enhanced water vapor permeation. Hence, it can be assumed that the presence of the Co–MOF may have altered the orientation of the polymer chains in the films, facilitating the easy penetration of water vapor. Although the increase in WVP may be considered a limitation from a conventional barrier perspective, this characteristic can be advantageous for intelligent packaging applications. The intrinsic porosity of Co–MOF structures facilitates the diffusion of volatile compounds, such as ammonia, through the film matrix, thereby enhancing the responsiveness and sensitivity of the colorimetric system. These findings suggest that increased water vapor permeability may contribute to the rapid response of the films during ammonia sensing.

### 3.2. Sorption Isotherms

The parameters for the water sorption isotherms of the G/Co–MOF composite films were analyzed using GAB and BET models, and the results are presented in Table 2. The results clearly demonstrate that both models provided an excellent description of the experimental moisture sorption behavior of G/Co–MOF composite films, as indicated by the high determination coefficients (R^2^ = 0.995–0.999). These high R^2^ values indicate a strong agreement between the experimentally measured equilibrium moisture content values and those predicted by the models, confirming the suitability of both approaches for describing water–film interactions. The BET and GAB models were used to obtain complementary information regarding the moisture sorption mechanisms of the composite films. From a physical perspective, the BET model describes water adsorption primarily through monolayer formation on active adsorption sites and provides valuable information about the monolayer moisture content (M_0_), which represents the amount of strongly bound water within the film matrix. However, BET does not consider the interactions occurring between multilayer water molecules. The GAB model extends the BET theory by incorporating multilayer adsorption and water–water interactions and therefore provides a more comprehensive description of moisture sorption behavior, particularly for food and biopolymer-based materials over a wider water activity range. Thus, the combined application of BET and GAB models enabled a more detailed evaluation of primary adsorption sites, multilayer water formation, and the overall water affinity of G/Co–MOF composite films.

The GAB and BET models indicated that the gelatin film without Co–MOF exhibited the lowest M_0_ values, whereas the incorporation of Co–MOF led to a marked increase in M_0_, indicating an increased number of available adsorption sites and enhanced moisture affinity of the composite films. This increase may be attributed to the high specific surface area and porous structure of Co–MOFs, which provide additional sites for water adsorption and contribute to the altered moisture sorption behavior of the films [28]. Similarly, both the Guggenheim constant (C) and BET constant (c) increased with increasing Co–MOF concentration, suggesting stronger interactions between water molecules and adsorption sites within the composite structure [29]. The increase in specific surface area from 356.13 to 455.74 m^2^/g further supports the enhanced moisture affinity observed after Co–MOF incorporation. Similar effects of Co–MOF incorporation on water adsorption behavior have also been reported by other researchers [15,30,31]. These findings highlight the potential of G/Co–MOF composite films for moisture-sensitive applications, including food packaging.

**Figure 1:**
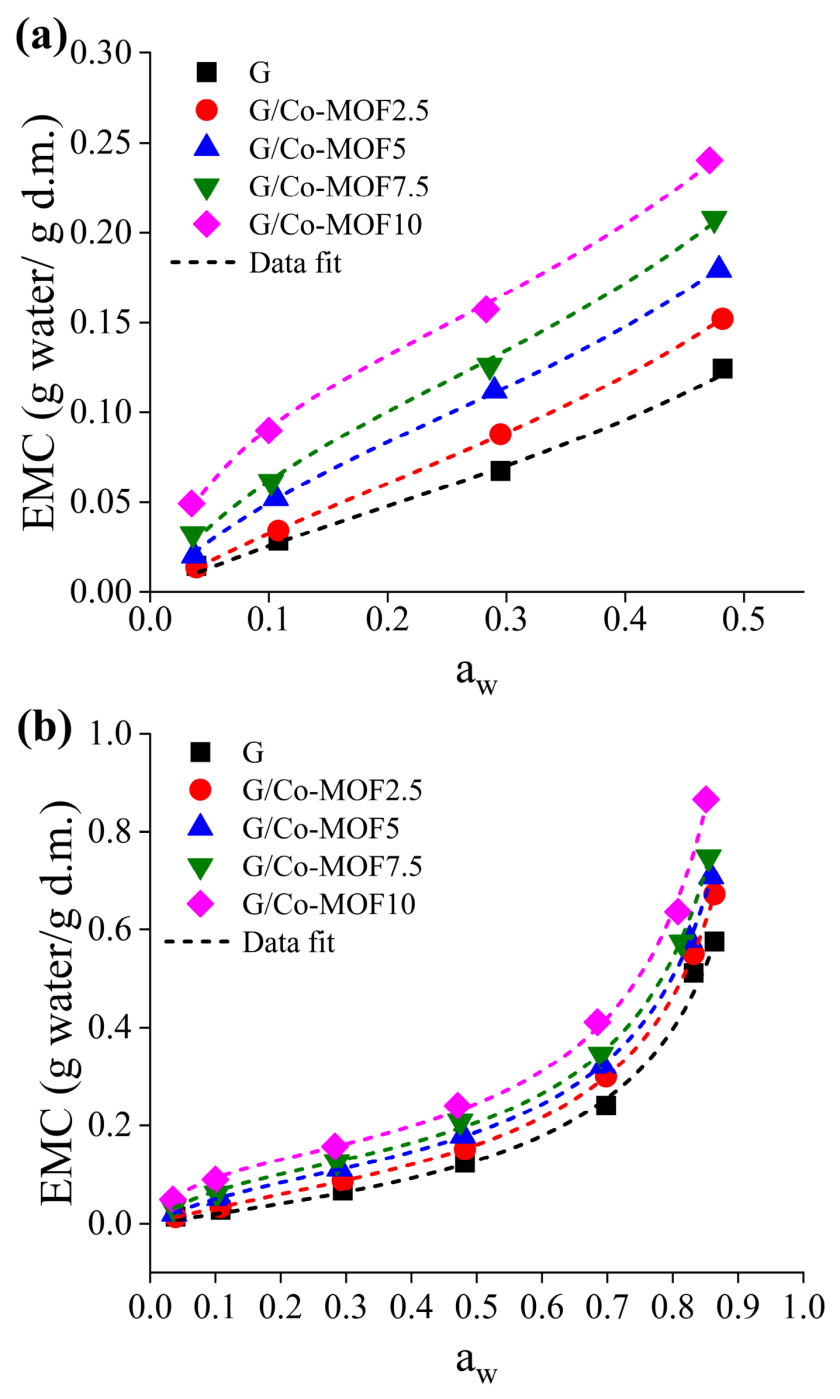
Moisture sorption isotherm curves with (**a**) GAB model and (**b**) BET model of G/Co–MOF composite films. G/Co–MOF0: Gelatin film without cobalt metal–organic framework; G/Co–MOF2.5: Gelatin film with 2.5% cobalt metal–organic framework; G/Co–MOF5: Gelatin film with 5% cobalt metal–organic framework; G/Co–MOF7.5: Gelatin film with 7.5% cobalt metal–organic framework; G/Co–MOF10: Gelatin film with 10% cobalt metal–organic framework. EMC: Equilibrium moisture content.

The moisture sorption isotherm curves with GAB (Figure 1a) and BET models (Figure 1b) of G/Co–MOF composite films are presented in Figure 1. In both models, equilibrium moisture content (EMC) increased progressively with water activity, indicating increased water uptake by the films. However, the sharper increase predicted by the BET model above aw 0.70 suggests intensified capillary condensation and water clustering phenomena at high humidity levels. The control gelatin film without Co–MOF showed the lowest EMC values in both models, whereas films containing Co–MOF exhibited progressively higher moisture uptake. In contrast, films containing Co–MOF exhibited progressively higher EMC values as the Co–MOF concentration increased, confirming that incorporating Co–MOF enhances the film’s hygroscopic behavior. This behavior could be attributed to the intrinsic porosity and high specific surface area of Co–MOF structures [32]. A recent study on MOF–polymer composites highlights that these porous architectures significantly enhance water sorption capacity and stability [33]. Moreover, comprehensive reviews have highlighted that tunable pore structures and modifiable surface chemistry of MOFs are key to tailoring water adsorption behavior in humid environments [30]. These findings confirm that Co–MOF incorporation enhances the water sorption capacity of gelatin films and may contribute to their functional performance in intelligent packaging applications.

### 3.3. Microstructural and Elemental Composition Analyses

**Figure 2:**
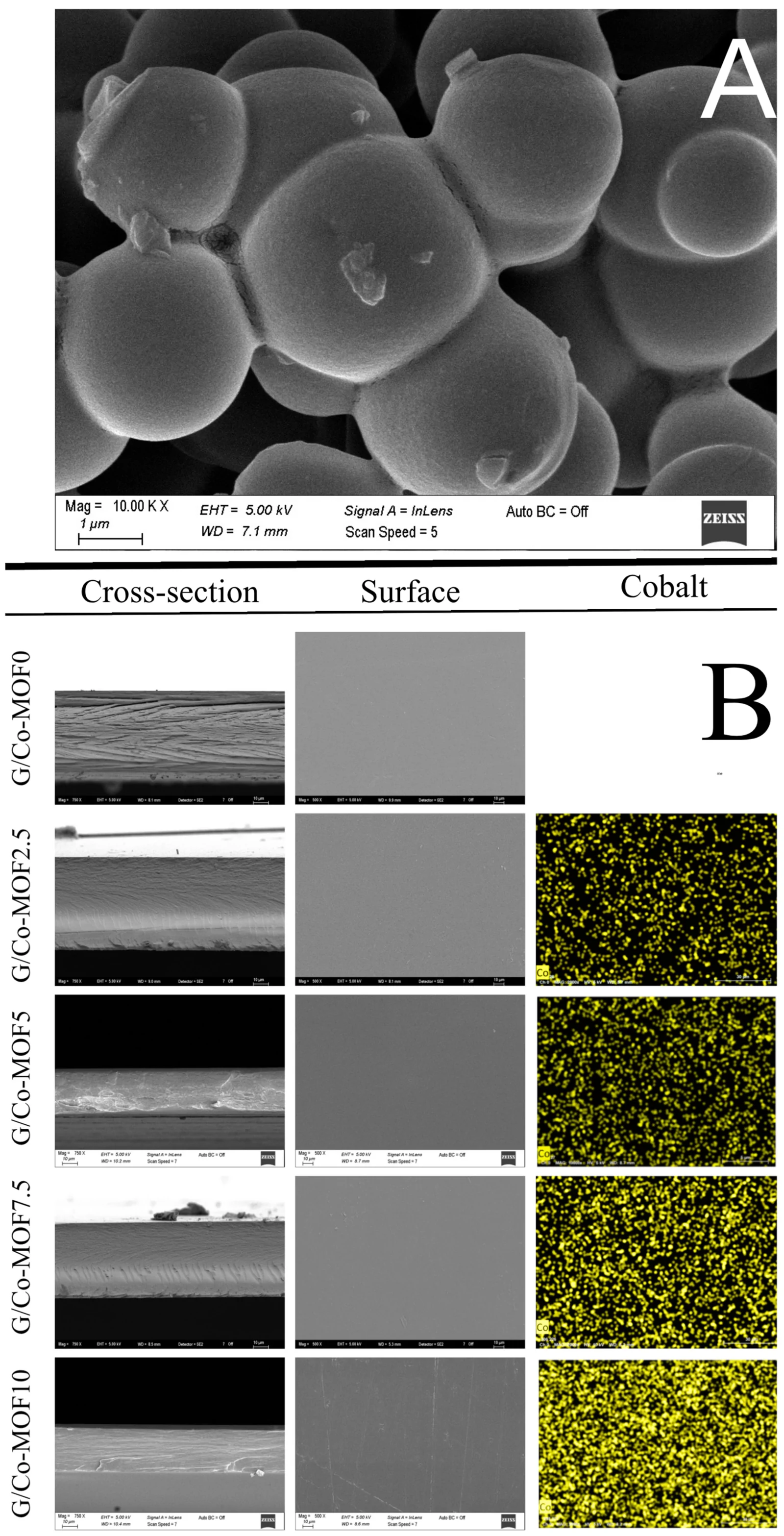
(**A**) SEM image of Co-MOF, (**B**) cross-sectional, surface, and elemental mapping images of G/Co–MOF composite films. G/Co–MOF0: Gelatin film without cobalt metal–organic framework; G/Co–MOF2.5: Gelatin film with 2.5% cobalt metal–organic framework; G/Co–MOF5: Gelatin film with 5% cobalt metal–organic framework; G/Co–MOF7.5: Gelatin film with 7.5% cobalt metal–organic framework; G/Co–MOF10: Gelatin film with 10% cobalt metal–organic framework (magnification of cross-section images is ×750 and of surface images is ×1000).

The microstructural analysis of gelatin-based films incorporating G/Co–MOFs at different loadings provides clear insight into how MOF incorporation influences both the internal morphology and surface characteristics of composite films. The microstructural properties and cobalt distribution of G/Co–MOF composite films are presented in Figure 2. SEM cross-sectional images revealed a progressive structural evolution with increasing MOF concentration. The control film (G/Co–MOF0) exhibited a dense, homogeneous matrix typical of plasticized gelatin structures, with no visible particulate domains or phase discontinuities. Upon the addition of 2.5% MOF, the cross-section maintained its overall compact architecture; however, very small dispersed domains began to appear, suggesting the early stages of MOF embedding within the polymer network without causing major morphological distortion. As the MOF content increased to 5% and 7.5%, the internal structures displayed more pronounced heterogeneities, with clearly distinguishable particulates and localized roughness within the matrix. These features indicate that the MOF crystals were successfully incorporated but micro-scale discontinuities and heterogeneous interfacial regions were generated, indicating the formation of localized heterogeneities within the polymer matrix. Despite this, the polymeric network remained largely intact, demonstrating that the gelatin matrix can accommodate moderate MOF loadings without severe structural disruption. At 10% MOF, the cross-section exhibited increased roughness and visible aggregates, reflecting the reduced dispersibility typical at higher filler concentrations. This suggests that excessive MOF addition can compromise the film’s structural uniformity, potentially affecting the physicochemical and functional properties of the films. As explained in the relevant sections, the negative effect of Co–MOF addition on the mechanical and barrier properties of the films, albeit slight, supports this idea. Similarly, Li et al. [8] reported that the membrane cross-section gradually becomes rougher after the addition of Cu-MOF to the chitosan/gelatin matrix.

Surface SEM images also reinforced these observations. The pristine gelatin film exhibited a smooth, featureless surface, confirming the absence of inorganic particulates, as also observed by Riahi et al. [5] and Khezerlou et al. [34]. Films containing 2.5–7.5% MOF retained a generally smooth morphology, although subtle textural variations emerged, likely associated with MOF particles embedded just beneath or slightly protruding from the surface. By contrast, the film containing 10% MOF showed the first clear evidence of surface irregularities, presumably due to particle agglomeration and limited polymer chain mobility around densely packed MOF structures. Similar results were also reported after adding metal-based MOFs, such as Cu-MOF [5,12], lactoferrin-loaded Cr-MOF [34], Zn-MOF [5], and Co–MOF [1,4,6] into the biopolymeric matrices. These changes indicate that the surface morphology became increasingly influenced by Co–MOF concentration. Cobalt elemental mapping provides further confirmation of MOF distribution throughout the film surface. At 2.5% and 5% MOF loadings, cobalt signals were uniformly distributed across the mapped regions, demonstrating effective dispersion of MOF particles within the gelatin matrix. This uniform distribution indicates good compatibility between the Co–MOF particles and the gelatin matrix at low and moderate loadings. Similarly, Li et al. [8] reported that low amounts of Cu-MOF (0.5, 1.0, and 1.5% w/w, based on gelatin) were uniformly embedded in the chitosan/gelatin matrix. However, in the 7.5% and 10% MOF-added films, cobalt mapping revealed areas of intensified signal density, as indicated by changes in color darkness, supporting the presence of locally concentrated MOF aggregates observed in SEM. Such clustering confirms the presence of localized Co–MOF aggregates at higher filler concentrations.

Furthermore, the elemental composition of the Co–MOF and G/Co–MOF composite films is presented in Table 3. Elemental analysis revealed the presence of C (38.72%), N (37.0%), O (4.75%), and Co (19.53%) in the composition of Co–MOF. These results are in good agreement with the expected stoichiometry of the synthesized Co–MOF and confirm the successful formation of the target material. However, Wang et al. [35] reported that the composition of Co–MOF synthesized using 4,4-diaminodiphenyl methane consisted of 83.2% C, 6.6% N, 7.0% O, and 3.2% Co. The composition of the pure gelatin film (G/Co–MOF0) consisted of 54.01% C, 17.24% N, and 28.77%, which is consistent with those reported by Mikhailov [36]. The elemental composition of G/Co–MOF films changed with the addition of Co–MOF, and while the C content increased to 60.62%, the O content decreased to 20.45% (*p* < 0.05), consistent with the elemental composition of Co–MOF. Co content increased with higher Co–MOF loading, from 0.68% at 2.5% to 1.66% at 10% (*p* < 0.05), confirming the successful integration of Co–MOF into the gelatin matrix. This morphological evolution is consistent with the observed mechanical and barrier properties, where increased aggregation at higher MOF loadings correlates with reduced tensile strength and increased water vapor permeability. These findings demonstrate that the dispersion state of Co–MOF particles significantly influences the structural, mechanical, and functional properties of the composite films.

### 3.4. Color, Opacity, and Visual Appearance

**Figure 3:**
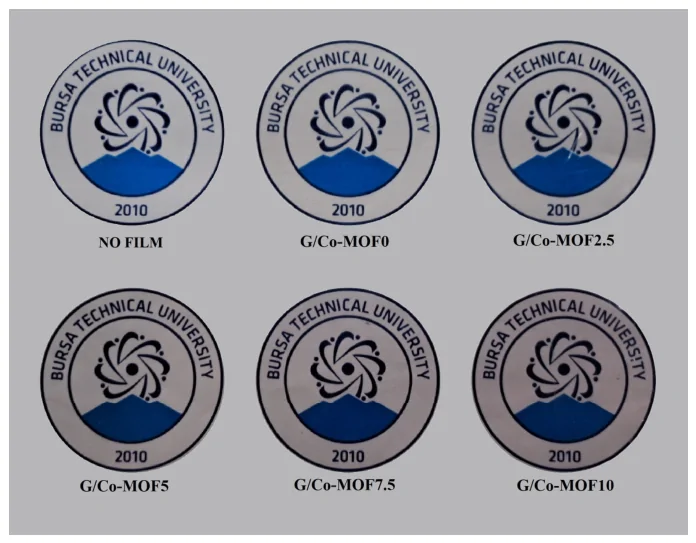
Visual appearances of G/Co–MOF composite films. G/Co–MOF0: Gelatin film without cobalt metal–organic framework; G/Co–MOF2.5: Gelatin film with 2.5% cobalt metal–organic framework; G/Co–MOF5: Gelatin film with 5% cobalt metal–organic framework; G/Co–MOF7.5: Gelatin film with 7.5% cobalt metal–organic framework; G/Co–MOF10: Gelatin film with 10% cobalt metal–organic framework.

The color and optical properties of gelatin and G/Co–MOF composite films are presented in Table 4, and the visual appearances are presented in Figure 3. Increasing the concentration of Co–MOF resulted in a decrease in the L* values from 96.10 to 94.51 and a reduction in the b* values from 1.75 to 0.68. This change led to the films appearing darker and less yellow. Conversely, the addition of Co–MOF increased the a* values; the a* value, which was −0.22 in the control group, rose to 1.65, giving the films a reddish hue. This behavior can be attributed to the presence of Co–MOF particles within the gelatin matrix, which influenced light absorption and scattering and consequently altered the optical appearance of the films. Yilmaz et al. [15] also reported that the addition of Co–MOF resulted in similar changes in the color values of the films. Additionally, the addition of Co–MOF resulted in a significant increase in opacity compared to the control gelatin film (*p* < 0.05). Specifically, the opacity increased from 1.159 for the control gelatin film without Co–MOF to 2.075, 2.107, and 2.199 for the films containing 5, 7.5, and 10 wt.% Co–MOF, respectively. The increase in opacity suggests that Co–MOF incorporation progressively reduced light transmission through the films because of the presence of dispersed inorganic particles within the polymer matrix. Similarly, previous reports also indicated that adding Co–MOF to the polymer matrix increased opacity [6].

As shown in Figure 3, the control gelatin film was relatively colorless and transparent. However, when Co–MOF was added, the films changed from colorless to rust red due to Co–MOF’s color, and the tint darkened with increasing Co–MOF content. Consequently, the transparency of films decreased, and they appeared darker and opaquer, especially at higher Co–MOF concentrations. This concentration-dependent optical response may be advantageous for intelligent packaging applications, where visible color changes facilitate visual interpretation of the sensing response. These findings confirm that Co–MOF incorporation significantly altered the optical properties of gelatin films and improved their visual response characteristics.

### 3.5. Thermal Properties

**Figure 4:**
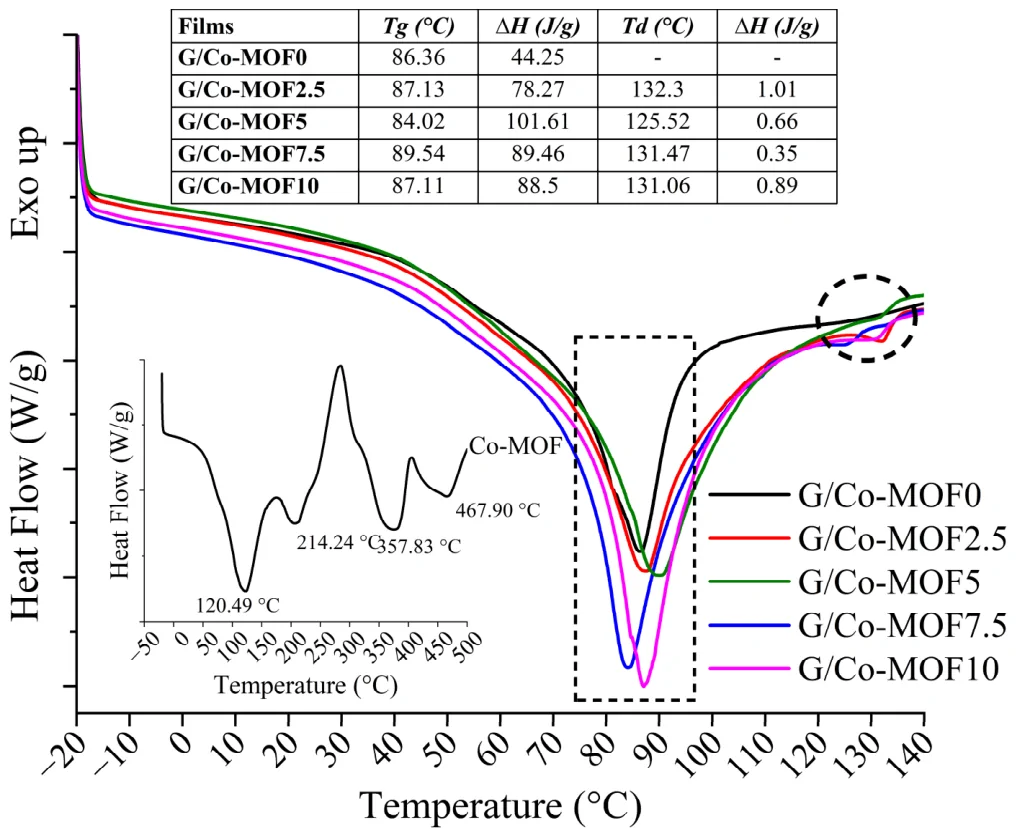
DSC thermograms of Co–MOF and G/Co–MOF composite films. Co–MOF: Cobalt metal–organic framework; G/Co–MOF0: Gelatin film without cobalt metal–organic framework; G/Co–MOF2.5: Gelatin film with 2.5% cobalt metal–organic framework; G/Co–MOF5: Gelatin film with 5% cobalt metal–organic framework; G/Co–MOF7.5: Gelatin film with 7.5% cobalt metal–organic framework; G/Co–MOF10: Gelatin film with 10% cobalt metal–organic framework.

Since the degradation behavior of packaging materials at high temperatures is crucial for industrial applications, especially during the manufacturing process, DSC analysis was conducted to evaluate the thermal stability of Co–MOF and G/Co–MOF composite films, and their thermograms are shown in Figure 4. The DSC curve of pristine Co–MOF exhibits multiple endothermic and exothermic events at approximately 120.5, 214.2, 357.8, and 467.9 °C, which can be attributed to the sequential removal of physically adsorbed water, decomposition of coordinated solvent molecules, and eventual breakdown of the MOF. These findings are also consistent with the observations of Ma et al. [33].

The pure gelatin film (G/Co–MOF0) displayed a clear thermal transition at around 86.36 °C, corresponding to the glass transition temperature (*Tg*) of gelatin. This transition is likely associated with the relaxation of gelatin molecular chains and the disruption of intermolecular hydrogen bonding within the amorphous regions. The relatively high enthalpy change (Δ*H* = 44.25 J/g) reflects the presence of ordered gelatin domains and strong interactions. Upon incorporation of Co–MOF into the gelatin matrix, some changes in *Tg* and enthalpy values were observed. At low Co–MOF loading (G/Co–MOF2.5), the *Tg* slightly increased to 87.13 °C, accompanied by a significant rise in Δ*H* (78.27 J/g). Interestingly, at moderate loading (G/Co–MOF5), a slight decrease in *Tg* (84.02 °C) was observed, while the enthalpy reached its maximum value (101.61 J/g). This behavior may be associated with good dispersion of Co–MOF particles within the gelatin matrix and interactions between the filler and the polymer network. As the Co–MOF content increased further (G/Co–MOF7.5 and G/Co–MOF10), the *Tg* values increased again (up to 89.54 °C), likely reflecting the influence of Co–MOF incorporation on the thermal behavior of the gelatin matrix. However, the associated Δ*H* values decreased compared to G/Co–MOF5, which may be attributed to partial aggregation of Co–MOF particles and reduced effective interaction area with gelatin chains.

Additionally, the appearance of a secondary thermal event (*Td*) in the range of ~125–132 °C for Co–MOF-containing films, which was absent in the pure gelatin film, suggests the occurrence of an additional thermal transition induced by Co–MOF incorporation. This event may be associated with interactions between Co–MOF particles and gelatin chains, which modify the local molecular environment and generate thermally distinguishable regions within the composite matrix. The emergence of this transition may be related to the restriction of gelatin chain mobility and the formation of additional polymer–MOF interaction regions, which alter the thermal response of the composite matrix. However, the relatively low ΔH values indicate that this transition involves only a limited fraction of the composite structure rather than representing a dominant thermal transition. The appearance of this secondary event, together with the changes in Tg and enthalpy values, confirms the influence of Co–MOF on the thermal behavior of gelatin films. The observed shifts in *Tg* and enthalpy changes, along with the emergence of additional thermal transitions, confirm strong interfacial interactions and improved thermal stability of the composite films. A similar study by Feng et al. [3] showed that adding Co–MOF to the sodium alginate matrix increased thermal resistance. The findings of Zhang et al. [7] also confirmed that the addition of Co–MOF to sodium carboxymethyl cellulose films improves thermal stability. These findings demonstrate that Co–MOF incorporation influences the thermal transition behavior of gelatin films and may contribute to their thermal resistance. Such effects may be beneficial for the development of intelligent packaging materials requiring adequate thermal resistance during processing and storage.

### 3.6. Crystallographic Properties

**Figure 5:**
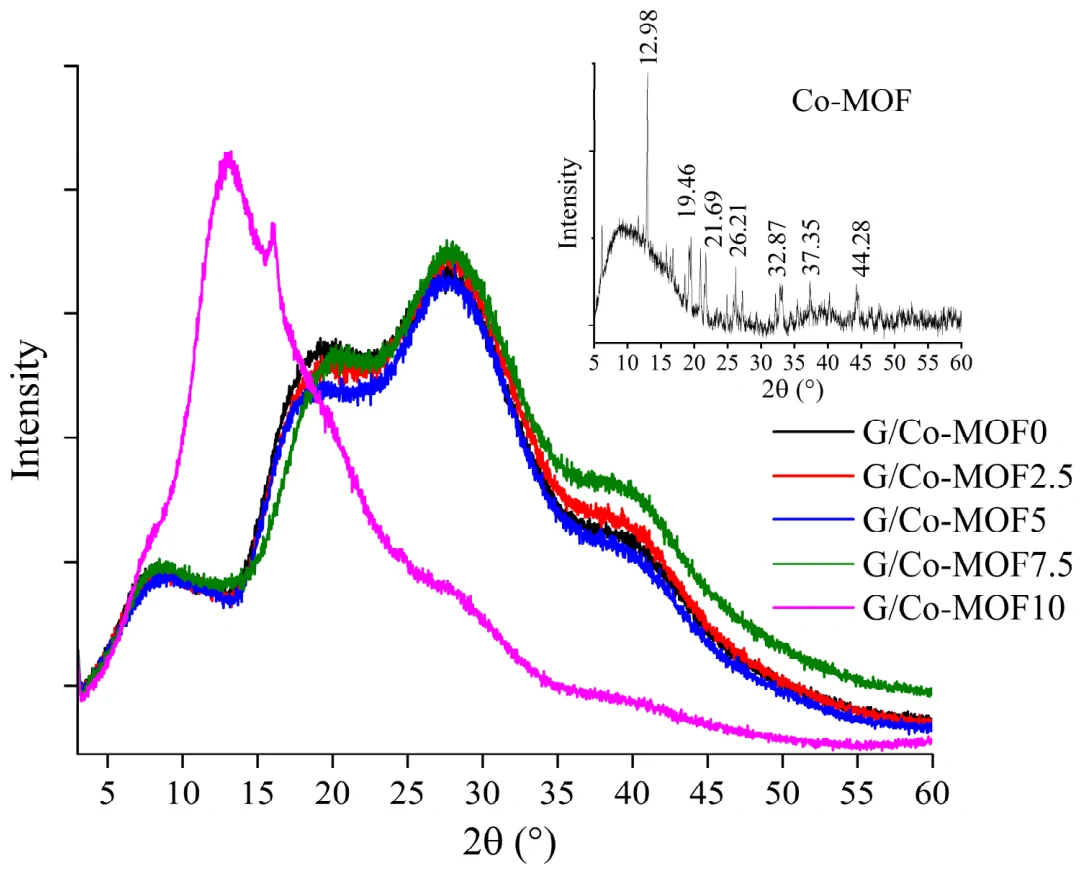
XRD patterns of Co–MOF and G/Co–MOF composite films. Co–MOF: Cobalt metal–organic framework; G/Co–MOF0: Gelatin film without cobalt metal–organic framework; G/Co–MOF2.5: Gelatin film with 2.5% cobalt metal–organic framework; G/Co–MOF5: Gelatin film with 5% cobalt metal–organic framework; G/Co–MOF7.5: Gelatin film with 7.5% cobalt metal–organic framework; G/Co–MOF10: Gelatin film with 10% cobalt metal–organic framework.

The X-ray diffraction (XRD) patterns of pristine Co–MOF, pure gelatin film, and gelatin-based composite films containing different amounts of Co–MOF are presented in Figure 5. The XRD pattern of pristine Co–MOF displayed several sharp and well-defined diffraction peaks at 2θ values of approximately 12.98, 19.46, 21.69, 26.21, 32.8°, 37.35, and 44.28°, confirming the formation of a highly ordered crystalline coordination framework [3]. These characteristic peaks are generally consistent with those reported for previously studied cobalt-based MOFs [37], indicating the successful formation of an ordered coordination network between Co ions and gallic acid.

In contrast, pure gelatin film (G/Co–MOF0) presented the characteristic XRD pattern of a partially crystalline material with four diffraction peaks around 2θ = 8, 20–22, 28, and 40°, which is typical of the semi-amorphous nature of gelatin. Similar broad peaks were widely reported for gelatin and other protein-based polymer films, arising from the random arrangement of polymer chains and the absence of long-range crystalline order [13,22,23]. Upon incorporation of Co–MOF into the gelatin matrix, no noticeable changes in the diffraction profiles were observed. For G/Co–MOF2.5 and G/Co–MOF5 films, the characteristic gelatin peaks remained detectable but showed reduced intensity and pronounced peak broadening. This behavior suggests that Co–MOF particles were well dispersed within the gelatin matrix and influenced the structural organization of the composite films. Interactions between gelatin functional groups and Co–MOF particles may contribute to the observed reduction in diffraction intensity and peak broadening [4,22,23]. When the Co–MOF content is increased to 7.5 wt% (G/Co–MOF7.5), a gradual increase in diffraction intensity in the 20–30° region is observed. This change suggests an increase in structural ordering within the composite films as the Co–MOF concentration increased. Nevertheless, the absence of prominent Co–MOF crystalline peaks suggests that the MOF particles are still strongly embedded in the polymer network. Similar behavior was reported for biopolymer composites reinforced with inorganic fillers, where increased filler content enhanced short-range ordering without inducing full crystallinity [5,22].

At the highest Co–MOF loading (G/Co–MOF10), a more pronounced diffraction feature appeared at low angles (around 10–15°), indicating the emergence of more ordered Co–MOF domains. This observation can be attributed to partial aggregation or the increased crystallite size of Co–MOF at high loadings, where the gelatin matrix becomes less capable of maintaining homogeneous filler. The more pronounced diffraction features observed for G/Co–MOF10 may also be attributed to the higher concentration of Co–MOF particles within the gelatin matrix. At lower concentrations, Co–MOF particles were more uniformly dispersed and their diffraction signals were partially masked by the predominantly amorphous gelatin structure. However, at 10% loading, increased particle–particle interactions and partial aggregation may have enhanced the detectability of Co–MOF crystalline domains, resulting in a more distinguishable diffraction pattern. This interpretation is also consistent with the SEM observations, which suggested the presence of localized particle aggregates at higher Co–MOF concentrations. Additionally, the increased inorganic content may contribute to the reduction in the amorphous character of the composite films. Similar trends were observed in polymer/MOF composites at high filler concentrations, where filler–filler interactions become dominant over polymer–filler interactions [31]. Overall, the XRD results confirm the successful incorporation of Co–MOF into the gelatin matrix and demonstrate that the structural organization of the composite films is strongly dependent on the Co–MOF content. Low and moderate MOF loadings preserve the predominantly amorphous nature of gelatin, while higher MOF concentrations induce partial crystallinity and enhanced structural ordering. These findings demonstrate that the structural organization of the composite films was influenced by Co–MOF concentration, with higher loadings promoting greater structural ordering within the gelatin matrix.

### 3.7. Molecular Characteristics

**Figure 6:**
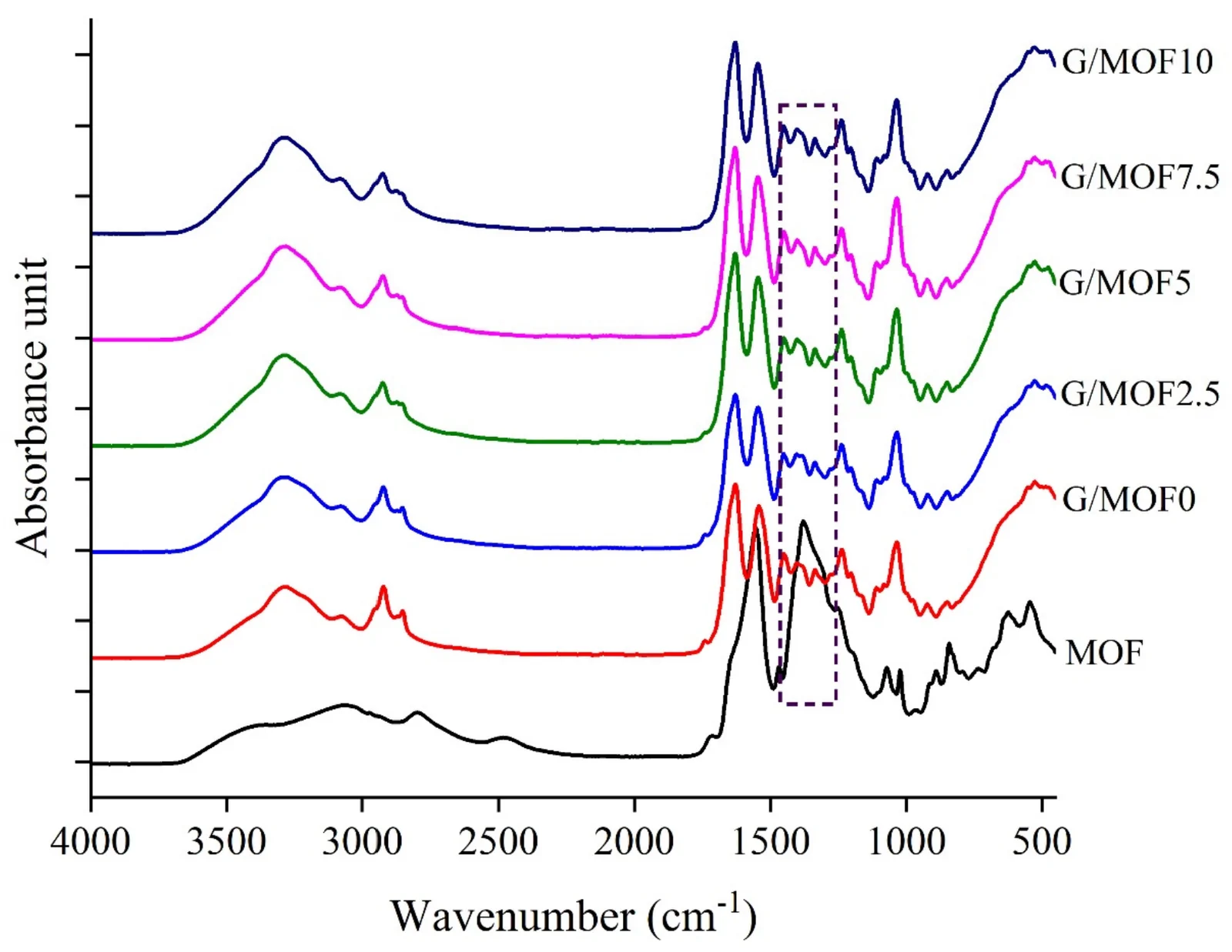
FTIR spectra of Co–MOF and G/Co–MOF composite films. Co–MOF: Cobalt metal–organic framework; G/Co–MOF0: Gelatin film without cobalt metal–organic framework; G/Co–MOF2.5: Gelatin film with 2.5% cobalt metal–organic framework; G/Co–MOF5: Gelatin film with 5% cobalt metal–organic framework; G/Co–MOF7.5: Gelatin film with 7.5% cobalt metal–organic framework; G/Co–MOF10: Gelatin film with 10% cobalt metal–organic framework.

The FTIR spectra of Co–MOF and gelatin-based composite films incorporating different loadings of Co–MOF (0–10 wt%) are presented in Figure 6. The FTIR spectrum of pure Co–MOF exhibited characteristic absorption bands in the low-wavenumber region (500–800 cm^−1^), which are attributed to Co-O stretching vibrations and metal–ligand coordination modes. Additionally, the strong bands observed in the range of approximately 1400–1600 cm^−1^ correspond to the asymmetric and symmetric stretching vibrations of coordinated carboxylate groups, confirming the formation of coordinated metal–ligand networks within the Co–MOF structure [7,38].

For the pure gelatin film (G/Co–MOF0), several typical absorption peaks were observed. The broad band around 3200–3400 cm^−1^ is associated with O-H and N-H stretching vibrations, indicating the presence of extensive hydrogen-bonding interactions within the gelatin matrix. The characteristic amide bands of gelatin were clearly visible: amide I (~1650 cm^−1^, C=O stretching), amide II (~1540 cm^−1^, N-H bending and C-N stretching), and amide III (~1230 cm^−1^), confirming the preservation of the protein backbone structure [39]. Upon incorporation of Co–MOF into gelatin, the composite films showed notable spectral changes. The amide I and amide II bands exhibited slight shifts and changes in intensity with increasing Co–MOF content, indicating the establishment of strong interfacial interactions and localized molecular reorganization within the composite matrix. These interactions may involve hydrogen bonding and coordination interactions between Co^2+^ centers and functional groups present in gelatin [7]. Moreover, the intensity of absorption bands in the 1000–1600 cm^−1^ region gradually increased with higher Co–MOF loading, reflecting the increasing contribution of MOF-associated vibrational bands as Co–MOF concentration increased [1]. The dashed region highlighted in the figure further emphasizes the gradual evolution of these bands, confirming the successful incorporation of Co–MOF into the gelatin matrix, in agreement with the SEM and XRD results. Importantly, no new peaks corresponding to chemical degradation or unwanted side reactions were observed. These findings suggest that Co–MOF incorporation influences molecular interactions within the gelatin matrix without affecting the integrity of the primary polymer backbone.

### 3.8. Mechanical Properties

The mechanical properties, including the TS, EAB, PF, and PD of G/Co–MOF composite films containing Co–MOF at different concentrations, are presented in Table 5. The TS value, determined as 59.47 MPa for the pure gelatin film, decreased with the addition of more than 5% Co–MOF, reaching 37.72 MPa in the composite films containing 10% Co–MOF (*p* < 0.05). This reduction is likely associated with the aggregation of Co–MOF particles within the polymer matrix, as observed in the SEM micrographs (Figure 2).

In addition, the reduction in film thickness may have contributed to the lower tensile strength values observed at high Co–MOF concentrations. In agreement with our findings, Riahi et al. [5] reported a decrease in TS upon adding 3% Zn-MOF to gelatin films, and Hong et al. [13] reported a decrease in TS upon adding 3% cinnamon essential oil-loaded MOF to gelatin/pullulan-based composite films. Despite this decrease in TS values, the addition of Co–MOF did not significantly change the EAB values of composite films (*p* > 0.05), consistent with a previously reported finding [22], suggesting that Co–MOF incorporation did not markedly affect the flexibility of the gelatin films. The SEM observations support the mechanical results, indicating that particle aggregation at high Co–MOF concentrations may negatively affect film performance. The PF and PD of G/Co–MOF composite films also decreased with the addition of Co–MOF, but this decrease was significant with the addition of more than 7.5% Co–MOF (*p* < 0.05) and reached 10.19 N and 2.41 mm, respectively, in films containing 10% Co–MOF. The reduction in puncture resistance at high MOF loadings may be associated with particle aggregation and less uniform filler distribution within the matrix. However, some studies [2,3,5] also suggest that MOF nanoparticles enhance mechanical properties. These contrasting findings indicate that the mechanical behavior of MOF-reinforced biopolymer films depends on the concentration, dispersion, and distribution of MOF particles within the polymer matrix.

### 3.9. Ammonia-Sensitivity Performance

**Figure 7:**
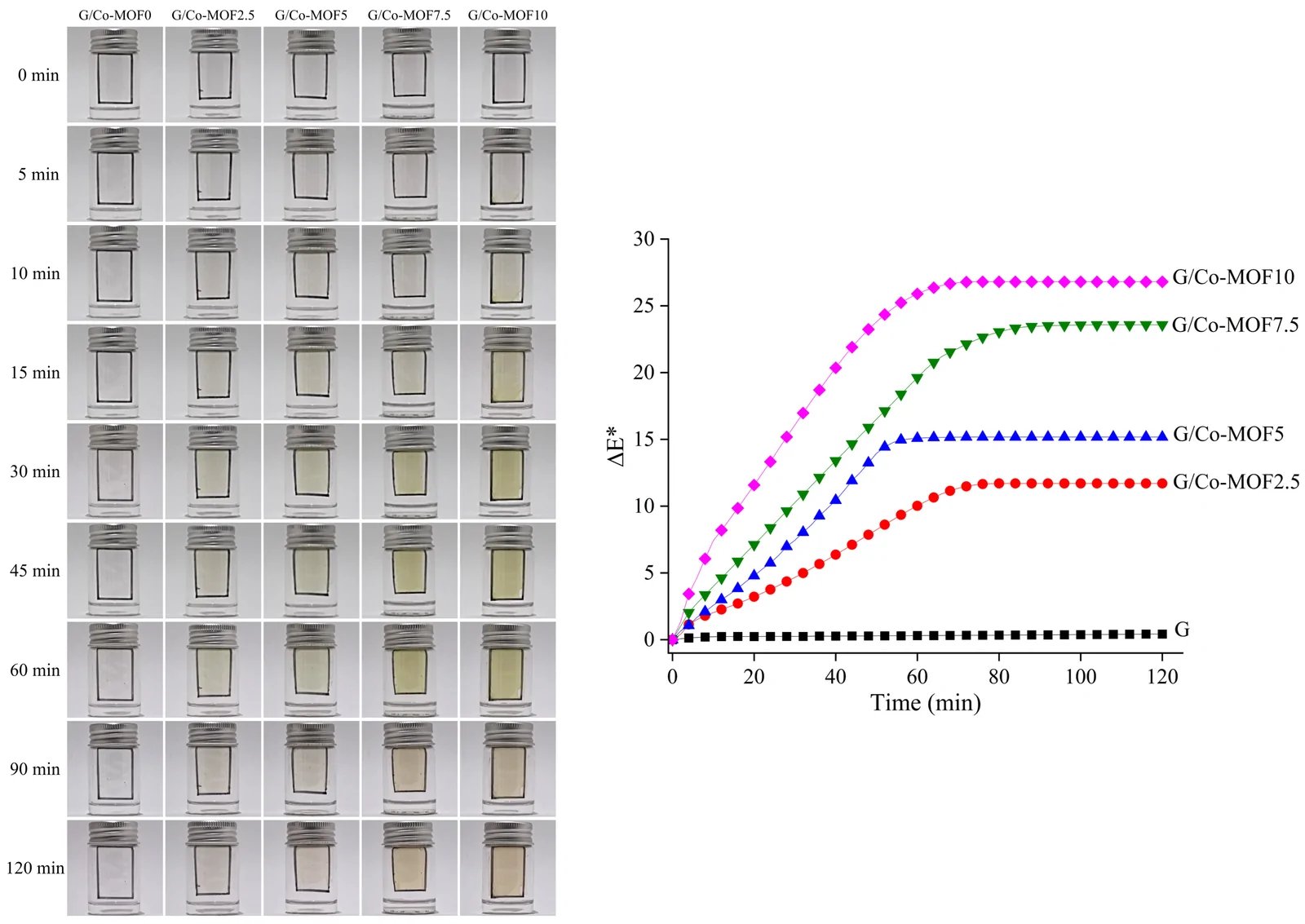
Ammonia-sensitivity performance of G/Co–MOF composite films. Visual appearance (on the left) and the color change (on the right) of the films during ammonia vapor exposure. G: Gelatin film without cobalt metal–organic framework; G/Co–MOF2.5: Gelatin film with 2.5% cobalt metal–organic framework; G/Co–MOF5: Gelatin film with 5% cobalt metal–organic framework; G/Co–MOF7.5: Gelatin film with 7.5% cobalt metal–organic framework; G/Co–MOF10: Gelatin film with 10% cobalt metal–organic framework.

The ammonia-sensitivity performance of the films was measured as the color change (ΔE*) resulting from exposure to ammonia vapor, and the results as a function of time are shown in Figure 7. The neat gelatin film showed almost no change in ΔE* value throughout the entire measurement period, indicating the absence of any significant chromatic response. In contrast, all G/Co–MOF composite films showed a pronounced and time-dependent increase in ΔE* value. Furthermore, the magnitude of ΔE* gradually increased with increasing Co–MOF concentration, indicating a concentration-dependent increase in ammonia sensitivity and color response. A similar relationship was also reported in thermoplastic starch films with Co–MOF incorporated by Yilmaz et al. [15]. The increase in color response with increasing Co–MOF concentration demonstrates the important contribution of Co–MOF particles to ammonia sensing performance. Additionally, it was observed that at 10 and 7.5% Co–MOF loading, the ΔE* value increased rapidly to values above 6 within 20 min, indicating a clearly distinguishable color change to the naked eye [1,15]. This rapid response is particularly relevant for real-time food freshness monitoring, as it enables early visual detection of spoilage-related volatile amines. This behavior may be attributed to the porous structure and high surface area of Co–MOF particles, which favor interactions with ammonia molecules. The colorimetric response of the G/Co–MOF films can be attributed to the interaction of ammonia molecules with the gallate-based Co–MOF structure. Previous studies have demonstrated that NH_3_ can strongly interact with gallate-based Co–MOFs through hydrogen-bonding interactions involving oxygen-containing functional groups as well as coordination interactions associated with cobalt centers [40]. Furthermore, ammonia is known to coordinate with cobalt species, altering their local electronic environment and consequently affecting optical properties, which may result in visible color changes [41]. Therefore, the observed color variation is likely associated with ammonia-induced modifications in the coordination and electronic structure of the Co–MOF domains embedded within the gelatin matrix. The shorter response time observed in the present system indicates a rapid response toward ammonia exposure compared with previously reported MOF-based intelligent films [1,15].

Also, it was observed that at 2.5% Co–MOF loading ΔE* increased gradually and reached approximately 11–12 after 70–80 min. A similar trend was observed for the 5% Co–MOF composite film. The steeper increase in ΔE* and earlier saturation behavior indicate improved ammonia sensing performance compared with the film containing 2.5% Co–MOF. Further increasing the Co–MOF concentration to 7.5% resulted in a substantial enhancement of color change, with ΔE* rising rapidly and stabilizing at around 23–24 after approximately 70 min. The highest color difference was observed for the G/Co–MOF composite film with 10% Co–MOF, which displayed the fastest response rate and the largest ΔE* value (~27). This sample reached saturation within approximately 60 min, indicating that higher Co–MOF concentrations enhanced the rate and intensity of the color response. The observed increase in ΔE* with increasing Co–MOF content can be attributed to the MOF’s higher density of active sites. Co–MOF structures likely provide a greater number of active sites for interaction with ammonia molecules, leading to more pronounced optical changes. These findings demonstrate that Co–MOF incorporation significantly improves the ammonia sensitivity of gelatin films, particularly at higher filler concentrations. The developed composite films exhibited rapid and clearly visible color changes, highlighting their potential as intelligent packaging materials for food freshness monitoring.

Although the developed films exhibited rapid and concentration-dependent colorimetric responses toward ammonia vapor, the present study primarily focused on demonstrating their feasibility as intelligent packaging indicators. Therefore, detailed sensor performance parameters such as the limit of detection, sensitivity, selectivity toward other volatile compounds, and interference effects were not evaluated. Future studies should investigate these parameters under controlled conditions to further assess the practical applicability of the developed films in real food packaging systems.

## 4. Conclusions

In this study, ammonia-responsive gelatin-based composite films were successfully developed by incorporating Co–MOFs synthesized using gallic acid as a naturally occurring phenolic organic ligand. The incorporation of Co–MOFs significantly influenced the physicochemical, structural, thermal, mechanical, and sensing properties of the gelatin films. Increasing Co–MOF content reduced moisture content and swelling behavior while enhancing solubility, water vapor permeability, and moisture sorption capacity. Sorption isotherm analyses based on GAB and BET models confirmed the improved water adsorption behavior and increased specific surface area of the composite films.

Morphological, crystallographic, and molecular analyses demonstrated that Co–MOFs were successfully incorporated into the gelatin matrix, showing relatively homogeneous dispersion at low and moderate loadings and partial aggregation at higher concentrations. FTIR, XRD, and DSC analyses indicated interactions between gelatin and Co–MOF particles and confirmed the improved thermal stability of the composite films. Although high Co–MOF loadings caused a reduction in tensile and puncture strength, the films retained adequate mechanical properties for packaging applications. Considering the balance between mechanical properties, barrier characteristics, and ammonia sensing performance, the G/Co–MOF7.5 film was identified as the most suitable formulation for intelligent packaging applications, providing enhanced sensing ability while maintaining acceptable structural integrity.

Most importantly, the gelatin/Co–MOF composite films exhibited rapid, concentration-dependent color changes upon exposure to ammonia vapor. Films containing higher amounts of Co–MOF showed faster response times and greater color differences, which were clearly distinguishable by the naked eye, demonstrating excellent ammonia-sensing performance. However, further studies are required to determine the detection limit, sensitivity, selectivity, and interference effects of the developed films before their practical implementation as ammonia-sensing indicators in commercial food packaging application. Overall, the results indicate that gallic acid-derived Co–MOFs represent promising functional fillers for the development of intelligent gelatin-based packaging materials. Furthermore, the use of gallic acid as a naturally occurring phenolic ligand may provide a more sustainable alternative to conventional MOF systems and may contribute to the development of intelligent packaging materials for food freshness monitoring applications. However, the developed Co–MOF films are intended for indirect freshness monitoring through ammonia vapor sensing, and further migration and safety evaluations are required before considering direct-food-contact applications.

## Figures and Tables

**Table 1:** Physicochemical and water vapor barrier properties of G/Co–MOF composite films.

Films	Physicochemical Properties				
	Thickness (mm)	Moisture Content (%)	Swelling Index (%)	Solubility (%)	WVP (g·mm/m^2^·h·kPa)
G/Co–MOF0	0.057 ± 0.002 ^a^	14.47 ± 0.33 ^a^	599.37 ± 24.36 ^a^	39.09 ± 0.29 ^e^	1.652 ± 0.008 ^e^
G/Co–MOF2.5	0.030 ± 0.002 ^d^	13.58 ± 0.28 ^b^	589.81 ± 9.05 ^ab^	41.72 ± 0.26 ^d^	1.763 ± 0.013 ^d^
G/Co–MOF5	0.030 ± 0.002 ^d^	13.44 ± 0.26 ^b^	484.88 ± 24.31 ^d^	42.98 ± 0.42 ^c^	1.832 ± 0.018 ^c^
G/Co–MOF7.5	0.045 ± 0.001 ^b^	13.37 ± 0.27 ^b^	547.30 ± 28.42 ^bc^	44.62 ± 1.22 ^b^	1.927 ± 0.013 ^b^
G/Co–MOF10	0.037 ± 0.002 ^c^	13.25 ± 0.11 ^b^	501.04 ± 14.64 ^cd^	48.88 ± 0.62 ^a^	2.054 ± 0.025 ^a^

Note: ^a–e^ Within each column, different lowercase superscripts indicate significant (*p* < 0.05) differences among film samples. G/Co–MOF0: Gelatin film without cobalt metal–organic framework; G/Co–MOF2.5: Gelatin film with 2.5% cobalt metal–organic framework; G/Co–MOF5: Gelatin film with 5% cobalt metal–organic framework; G/Co–MOF7.5: Gelatin film with 7.5% cobalt metal–organic framework; G/Co–MOF10: Gelatin film with 10% cobalt metal–organic framework. WVP: Water vapor permeability.

**Table 2:** Model parameters of water sorption isotherm data of G/Co–MOF composite films.

Model Parameters	Model Values	Films				
		G/Co–MOF0	G/Co–MOF2.5	G/Co–MOF5	G/Co–MOF7.5	G/Co–MOF10
GAB	$M_{0}^{1}$	0.102	0.107	0.109	0.115	0.130
	$c_{1}$	1.99	3.34	6.46	9.75	16.45
	k	0.970	0.982	0.989	0.992	0.997
	$S_{0}$	356.13	376.04	382.87	401.56	455.74
	$R^{2}$	0.995	0.999	0.999	0.999	0.997
BET	$M_{0}^{2}$	0.082	0.103	0.111	0.125	0.137
	$c_{2}$	3.50	3.57	6.09	7.26	13.50
	$R^{2}$	0.995	0.999	0.999	0.997	0.998

Note: G/Co–MOF0: Gelatin film without cobalt metal–organic framework; G/Co–MOF2.5: Gelatin film with 2.5% cobalt metal–organic framework; G/Co–MOF5: Gelatin film with 5% cobalt metal–organic framework; G/Co–MOF7.5: Gelatin film with 7.5% cobalt metal–organic framework; G/Co–MOF10: Gelatin film with 10% cobalt metal–organic framework. GAB: Guggenheim–Andersen–de Boer. $M_{0}^{1}$: Monolayer moisture content (MC) determined with the GAB equation in g H_2_O/g dry solid, $c_{1}$: Guggenheim constant related to the monolayer heat sorption, k: constant related to the multilayer heat sorption, $S_{0}$: specific surface area of films, $R^{2}$: coefficient of determination, BET: Brunauer–Emmett–Teller, $M_{0}^{2}$: monolayer MC determined with the BET equation in g H_2_O/g dry solid, $c_{2}$: BET constant related to the first layer heat of sorption.

**Table 3:** Elemental composition of Co–MOF and G/Co–MOF composite films.

Films	Elements			
	Carbon (%)	Nitrogen (%)	Oxygen (%)	Cobalt (%)
Co–MOF	38.72 ± 0.72 ^e^	37.00 ± 0.24 ^a^	4.75 ± 0.05 ^e^	19.53 ± 0.48 ^a^
G/Co–MOF0	54.01 ± 0.31 ^d^	17.24 ± 0.19 ^b^	28.77 ± 0.12 ^a^	0 ± 0
G/Co–MOF2.5	56.80 ± 0.06 ^c^	17.98 ± 0.35 ^b^	24.55 ± 0.25 ^b^	0.68 ± 0.04 ^e^
G/Co–MOF5	59.83 ± 0.33 ^b^	17.26 ± 0.25 ^b^	21.84 ± 0.54 ^c^	1.08 ± 0.03 ^d^
G/Co–MOF7.5	61.03 ± 0.11 ^a^	17.32 ± 0.08 ^b^	20.41 ± 0.23 ^d^	1.24 ± 0.03 ^c^
G/Co–MOF10	60.62 ± 0.23 ^a^	17.28 ± 0.13 ^b^	20.45 ± 0.08 ^d^	1.66 ± 0.01 ^b^

Note: ^a–e^ Within each column, different lowercase superscripts indicate significant (*p* < 0.05) differences among film samples. Co–MOF: Cobalt metal–organic framework. G/Co–MOF0: Gelatin film without cobalt metal–organic framework; G/Co–MOF2.5: Gelatin film with 2.5% cobalt metal–organic framework; G/Co–MOF5: Gelatin film with 5% cobalt metal–organic framework; G/Co–MOF7.5: Gelatin film with 7.5% cobalt metal–organic framework; G/Co–MOF10: Gelatin film with 10% cobalt metal–organic framework.

**Table 4:** Color and optical properties of G/Co–MOF composite films.

Films	Color Properties			Opacity
	L*	a*	b*	
G/Co–MOF0	96.10 ± 0.33 ^a^	−0.22 ± 0.04 ^e^	1.75 ± 0.23 ^a^	1.159 ± 0.035 ^c^
G/Co–MOF2.5	95.63 ± 0.23 ^b^	0.54 ± 0.02 ^d^	1.31 ± 0.06 ^b^	1.843 ± 0.121 ^b^
G/Co–MOF5	95.52 ± 0.27 ^b^	0.73 ± 0.10 ^c^	0.89 ± 0.25 ^c^	2.075 ± 0.214 ^a^
G/Co–MOF7.5	94.88 ± 0.46 ^c^	1.65 ± 0.13 ^a^	0.68 ± 0.29 ^c^	2.107 ± 0.012 ^a^
G/Co–MOF10	94.51 ± 0.09 ^c^	1.46 ± 0.16 ^b^	0.70 ± 0.08 ^c^	2.199 ± 0.078 ^a^

Note: ^a–e^ Within each column, different lowercase superscripts indicate significant (*p* < 0.05) differences among film samples. G/Co–MOF0: Gelatin film without cobalt metal–organic framework; G/Co–MOF2.5: Gelatin film with 2.5% cobalt metal–organic framework; G/Co–MOF5: Gelatin film with 5% cobalt metal–organic framework; G/Co–MOF7.5: Gelatin film with 7.5% cobalt metal–organic framework; G/Co–MOF10: Gelatin film with 10% cobalt metal–organic framework.

**Table 5:** Mechanical properties of G/Co–MOF composite films.

Films	Mechanical Properties			
	Tensile Strength (MPa)	Elongation at Break (%)	Puncture Force (N)	Puncture Deformation (mm)
G/Co–MOF0	59.47 ± 1.75 ^a^	112.06 ± 2.98	18.03 ± 1.39 ^a^	3.01 ± 0.26 ^a^
G/Co–MOF2.5	58.28 ± 2.91 ^a^	111.43 ± 1.23	17.99 ± 1.55 ^a^	2.96 ± 0.17 ^a^
G/Co–MOF5	51.20 ± 2.71 ^b^	110.81 ± 1.17	17.73 ± 0.69 ^a^	2.81 ± 0.21 ^a^
G/Co–MOF7.5	41.61 ± 2.71 ^c^	110.01 ± 1.07	13.70 ± 1.47 ^b^	2.52 ± 0.12 ^b^
G/Co–MOF10	37.72 ± 2.30 ^d^	107.69 ± 3.05	10.19 ± 0.24 ^c^	2.41 ± 0.04 ^b^

Note: ^a–d^ Within each column, different lowercase superscripts indicate significant (*p* < 0.05) differences among film samples. G/Co–MOF0: Gelatin film without cobalt metal–organic framework; G/Co–MOF2.5: Gelatin film with 2.5% cobalt metal–organic framework; G/Co–MOF5: Gelatin film with 5% cobalt metal–organic framework; G/Co–MOF7.5: Gelatin film with 7.5% cobalt metal–organic framework; G/Co–MOF10: Gelatin film with 10% cobalt metal–organic framework.

## Data Availability

The original contributions presented in this study are included in the article. Further inquiries can be directed to the corresponding authors.
